# Endomyocardial Fibrosis: Still a Mystery after 60 Years

**DOI:** 10.1371/journal.pntd.0000097

**Published:** 2008-02-27

**Authors:** Gene Bukhman, John Ziegler, Eldryd Parry

**Affiliations:** 1 Division of Social Medicine and Health Inequalities, Brigham and Women's Hospital, Boston, Massachusetts, United States of America; 2 Cancer Risk Program, University of California San Francisco, San Francisco, California, United States of America; 3 Clinical Research Unit, London School of Hygiene & Tropical Medicine, London, United Kingdom; Ghana Health Service, Ghana

## Abstract

The pathologist Jack N. P. Davies identified endomyocardial fibrosis in Uganda in 1947. Since that time, reports of this restrictive cardiomyopathy have come from other parts of tropical Africa, South Asia, and South America. In Kampala, the disease accounts for 20% of heart disease patients referred for echocardiography. We conducted a systematic review of research on the epidemiology and etiology of endomyocardial fibrosis. We relied primarily on articles in the MEDLINE database with either “endomyocardial fibrosis” or “endomyocardial sclerosis” in the title. The volume of publications on endomyocardial fibrosis has declined since the 1980s. Despite several hypotheses regarding cause, no account of the etiology of this disease has yet fully explained its unique geographical distribution.

September 2007 will mark the 60th anniversary of the description of endomyocardial fibrosis (EMF) in Uganda by the pathologist Jack N. P. Davies [1]. Observed by Arthur Williams as early as 1938, Davies and his colleagues at Makerere University delineated the clinico-pathologic features of this new restrictive cardiomyopathy, still called Davies disease by some [2],[3],[4]. Although virtually unknown outside of the tropics, cases of EMF continue to surface from parts of equatorial Asia and South America where the disease afflicts impoverished children and young adults [5]. The highest prevalence of this condition likely remains, however, in regions of sub-Saharan Africa. As a rough estimate, the burden of EMF may well compare in scope to Chagas cardiomyopathy [6].

Subendocardial fibrosis of the apices and inflow tracts of the right ventricle, left ventricle, or both defines the disease [7],[8]. This restrictive scarring prevents ventricular filling, and tethering of the papillary muscles leads to valvular regurgitation (Figure 1; Video S1). A review of autopsies in Uganda between 1959 and 1969 emphasized the poor prognosis of this condition, with an average survival of 2 y after symptom onset [9]. Later series from Brazil and India found more variability in the course of medically treated patients and echoed findings from southern Nigeria of both acute and chronic forms of the disease [10],[11],[12]. The advent of surgical resection and valvular replacement during the 1970s promised 10-y survival rates as high as 68% for selected patients, but at the price of high peri-operative mortality [13],[14],[15],[16],[17]. Unfortunately, EMF has most affected those regions least equipped with cardiovascular surgery.

**Figure 1:**
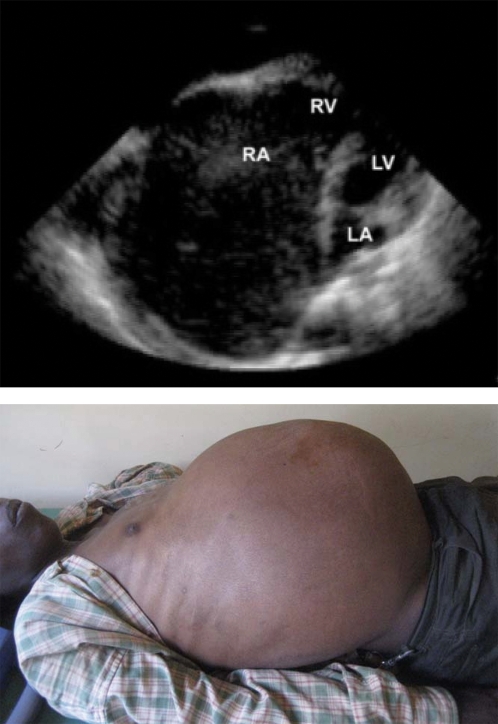
Top, echocardiogram in a 25-y-old man with predominantly right ventricular EMF from eastern Rwanda. Apical four-chamber view. Note the marked dilatation of the right atrium. RV = right ventricle, RA = right atrium, LV = left ventricle, LA = left atrium. Bottom, massive ascites in the same patient.

The question of whether all cases of EMF have the same underlying cause still ranks as one of the great mysteries in cardiology. Does the pathogenesis of this disease result from a single process? Or does EMF represent a common pathway for diverse insults such as those that lead to dilated cardiomyopathies?

Davies himself, who died in 1998 at the age of 83, believed to the end that EMF had a unifying explanation [18]. He thought the clue perhaps lay in the similarity between the heart lesion in EMF and the *endocarditis parietalis fibroplastica* that Wilhelm Löffler and others had described in Europe in the setting of hypereosinophilic syndromes [19],[20]. The eosinophil hypothesis—dominant though still not well tested—has failed to convince critics who point to other plausible alternatives [21],[22],[23],[24]. In fact, none of the etiologic categories first mentioned by Williams, Ball, and Davies in 1954 have left the table of possible causes (Table 1) [25].

**Table 1:** Proposed Causes of Endomyocardial Fibrosis.

Cause		Reference
**Infection**	Toxoplasmosis	[85]
	Rheumatic fever	[39],[86]
	Malaria	[87],[88]
	Myocarditis	[89]
	Helminthic parasites	[46],[62]
**Allergy**	Eosinophilia	[90]
	Auto-immunity	[76],[88]
**Malnutrition**	Protein deficiency	[79]
	Magnesium deficiency	[23]
**Toxic agents**	Cerium	[23]
	Cassava	[79],[91]
	Thorium	[23]
	Serotonin	[50]
	Plant toxin	[92]
	Vitamin D	[91]

Despite uncertainty as to the cause of EMF, the volume of publications on the subject has declined during the past decade (Figure 2). In an effort to rekindle interest in this neglected disease, we have undertaken a systematic review of research on this condition. We have based this review primarily on articles in the MEDLINE database published between January 1, 1950 and January 1, 2007 with either “endomyocardial fibrosis” or “endomyocardial sclerosis” in the title. We limited this search to articles in English, French, or Spanish and did not search other databases. We consulted additional papers and books referenced through this search strategy, and have cited those most focused on epidemiology and etiology.

**Figure 2:**
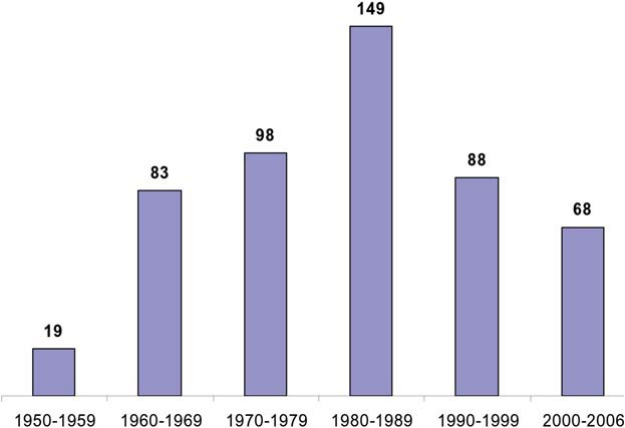
Number of publications in MEDLINE between 1950 and 2006 with either “endomyocardial fibrosis” or “endomyocardial sclerosis” in the title.

## Epidemiology

The clinical manifestations of EMF of either ventricle overlap with other conditions that cause heart failure or ascites. For this reason, a conclusive diagnosis of EMF depends on imaging or surgical visualization of the heart during life, or on autopsy after death [26],[27],[28].

**Figure 3:**
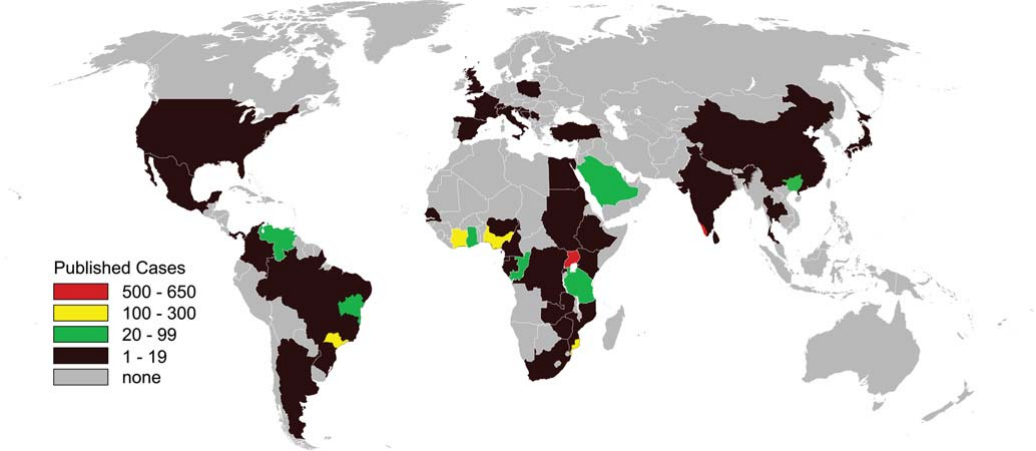
Distribution by country of published cases of endomyocardial fibrosis between 1950 and 2006. Includes only those cases diagnosed at autopsy, or confirmed by surgery or cardiac imaging. Within-country variation depicted for Brazil, China, India, Mozambique, and Nigeria.

Since the first descriptions of EMF at autopsy in West and East Africans in the late 1940s, over 2,400 cases of the disease have been reported throughout the world [1],[29]. Half of these cases have come from sub-Saharan Africa, and a quarter have come from Uganda alone. Connor and colleagues have questioned the relationship between Ugandan EMF and the West African disease [3]. Other regions with large series include Brazil, Côte d'Ivoire, southern Nigeria, coastal Mozambique, and Kerala State in India (Figure 3). Reporting bias skews this distribution, and in the absence of population-based studies, worldwide prevalence can only be estimated.

The frequency of EMF cases in Uganda has a bimodal peak at age 10 and age 30 [30]. Childhood EMF in this country affects boys and girls equally, while adult EMF affects women twice as often as men [30],[31]. In Nigeria, some studies have found a two to one male preponderance, while others have not shown any difference between the sexes [32].

The majority of EMF cases have come from low-lying, humid parts of tropical countries (Table 2). In East Africa, Uganda has a striking burden of EMF in contrast with Kenya and the Ethiopian highlands. In Tanzania and Mozambique, cases have clustered along the coastal forest [33],[34],[35]. Despite the frequency of EMF in the areas around the southern cities of Ibadan and Enugu in Nigeria, a review of cardiovascular admissions to a referral center in Zaria's northern savanna during the 1970s found no patients with this disease [36],[37],[38],[39]. In India, Kerala's tropical rain forest has generated one of the largest case series in the world, while other parts of the country have reported relatively few cases. In China, the largest number of case reports has also come from the southern province of Guangxi [40]. In South America, patients with EMF have come from Brazil and Columbia rather than Peru or Ecuador.

**Table 2:** Prevalence of EMF in Africa, Latin America, South Asia, China, and the Middle East.

	Authors	Country	City or Region	Dx^a^	Dates	Pop^b^	*n*	Ages	Set^c^	EMF
**Sub-Saharan Africa**	Freers et al. [42]	Uganda	Kampala	E	’93–94	CV	500	All	O	20%
	Williams et al. [25]	Uganda	Kampala	N	’51–53	HF	231	All	I	15%
	Brockington and Edington [90]	Nigeria	Ibadan	N	’58–66	CV	252	All	I	16%
	Abrahams [93]	Nigeria	Ibadan	A,N	’62	—	—	—	—	“common”
	Nwokolo [94]	Nigeria	Enugu	N	’58^d^	CV	—	All	I	“<5%”
	Betrand et al. [95]	Côte d'Ivoire	Abidjan	A,N	’75^d^	HF	—	< 40	I	25%
	Amoah et al. [96]	Ghana	Accra	E	’92–95	HF	572	Adult	I	4%
	Kimbally-Kaky [97]	Congo	Brazzaville	E	’88–00	HF	2,530	Adult	I	1%
	Turner and Manson-Bahr [98]	Kenya	Nairobi	N	’57–58	—	—	—	—	“rare”
	Kingue et al. [99]	Cameroon	Yaoundé	E	’98–01	HF	177	Adult	I	0%
	Maru [100]	Ethiopia	Addis Ababa	E	’85–88	CV	474	All	O	0%
	Daniel and Abegaz [101]	Ethiopia	Addis Ababa	E	’89–92	CV	468	< 18	O	0%
	Hodes [102]	Ethiopia	Addis Ababa	E	’85–86	CV	338	> 12	O	0%
	Harling et al. [103]	Gambia	Fajara	N	’61	CV	34	All	I	0%
	Oyoo and Ogola [104]	Kenya	Nairobi	E	’93	CV	91	Adult	I	0%
	Diallo et al. [105]	Mali	Bamako	E	’00–02	HF	436	Adult	I	0%
	Thiam [106]	Senegal	Dakar	E	’01	HF	170	Adult	I	0%
	Steenekamp et al. [107]	South Africa	Kelksdorp	N	’89	CV	74	All	I	0%
	Richter et al. [108]	Sudan	Wad Medani	E	’87	CV	33	All	I	0%
**Latin America**	Guimaraes [109]	Brazil	Bahia	N	’70–91	CV	734	All	I	2%
	Suarez and Suarez [110]	Venezuela	Caracas	N	’73^d^	—	—	—	—	“rare”
	Christie (L. Christie, personal communication, 2006)	Haiti	Deschapelles	E	’94–06	CV	—	All	O,I	“none”
**South Asia**	Kutty et al. [78]	India	Trivandrum	E	’78–94	CV	22,666	All	O	1.5%
	Datta and Aikat [111]	India	Chandigarh	N	’64–72	CV	906	All	I	0.9%
	Cherian et al. [16]	India	Chennai	S	’06^d^	—	—	—	—	“rare”
**China**	Yin et al. [40]	China	Guangxi	E	’00^§d^	CMP	—	All	I	“3%”
**Middle East**	Rashwan et al. [112]	Egypt	Alexandria	E	’91–93	CV	10,000	All	O	0.2%

a Dx = diagnostic modality, A = angiography, E = echocardiography, N = necropsy, S = surgery.

b Pop = population, CMP = only cardiomyopathy, CV = all patients with cardiovascular disease, HF = only heart failure.

c Set = setting, I = inpatient, O = outpatient.

d Year of publication.

## Etiology

Theories about the etiology of EMF have tried to explain the condition's unusual geography and pathology. The apparent concentration of EMF in the tropics has led to a search for infectious or nutritional causes. In particular, the similarity of EMF lesions to those in Löffler endocarditis and carcinoid heart disease has suggested a connection with serotonin or eosinophil toxicity.

Unfortunately, research on EMF peaked prior to the diffusion of echocardiography in the much of the tropics [28],[41],[42],[43],[44]. The lack of non-invasive imaging restricted studies to small autopsy or angiocardiographic series [45]. Descriptions of the clinical progression of the disease suffered from lack of diagnostic confirmation as well [12],[46]. Expansion of echocardiographic referral centers and the development of a sub-Saharan heart failure registry will do much to clarify the epidemiology of EMF in this region [47].

At present, only a few investigators have tested the proposed causes of EMF. Early enthusiasm for the role of serotonin in a plantain-based diet waned by the early 1970s [48],[49],[50]. Encouraged at first by the demonstration of high 5-hydroxyindole-acetic acid (5-HIAA) levels in the urine of West and East Africans, this work culminated when McKinney and Crawford fed plantains to guinea pigs, rats, and Patus monkeys [49],[51],[52],[53],[54]. They could not reproduce typical EMF lesions. With the finding that serum 5-hydroxytryptamine (5-HT) levels failed to rise in EMF patients fed a diet of plantains in Nigeria, investigation on this hypothesis ceased [55].

The eosinophil hypothesis gained prominence in the 1960s with reports of eosinophilic endomyocardial disease among European visitors to tropical regions [56],[57],[58],[59],[60],[61]. At the same time, Ive and Brockington in Nigeria found filariasis (onchocerciasis or loiasis) rates approaching 100% among 42 patients with angiographic EMF compared with 44% of 115 controls (*p*<0.001) [62],[63]. The suggestion that helminth-induced eosinophilia precipitated a tropical variant of the eosinophilic heart disease known as Löffler's found support in later accounts of helminth-associated EMF in natives and visitors to sub-Saharan Africa [64],[65].

The case for the equivalence of end-stage Löffler's and EMF rests on two formal evaluations [31]. The first study, published by Brockington and Olsen in 1975, compared the histology of 30 cases of Löffler's with 32 cases of EMF drawn from Uganda, Nigeria, and Brazil [66]. On the basis of this work, Olsen proposed three stages of Löffler's [67]. In patients with 1 to 2 mo of symptoms prior to autopsy, an eosinophilic myocarditis marked the necrotic stage. Those who died after 10 mo of symptoms had endocardial thickening and thrombosis rather than myocarditis. Among those 16 Löffler patients with more than 2 y of symptoms prior to autopsy, Olsen described a final fibrotic stage that he found identical to EMF. In a clinical and echocardiographic study published in 1983, Davies and colleagues confirmed these findings [31]. In 11 patients from the United Kingdom on the one hand, and 47 patients from India and Brazil on the other, they found no significant differences between fibrotic stage Löffler's and EMF.

Endomyocardial biopsies have failed, however, to demonstrate an eosinophilic myocarditis in EMF. In a series of 49 patients with EMF who underwent biopsies in Uganda, none had tissue eosinophilia despite early presentation in several cases [68]. Attempts to reproduce the Nigerian filariasis findings in other small studies have also failed to show a difference in prevalence of parasite exposure or eosinophilia between EMF cases and controls [69],[70]. In Uganda, one study found that 60% of echocardiographic cases had at least mild eosinophilia compared with 10% of controls (odds ratio 4.6) [30]. Another small study from this country has not shown a difference in rates of eosinophilia [24].

More recently, Andy and colleagues in Nigeria have argued in favor of the helminth-driven eosinophilia hypothesis. In a fascinating study weakened somewhat by lack of diagnostic confirmation, the investigators found an inverse relationship between eosinophil levels and duration of EMF disease [46]. In 89 cases of EMF, only 20% of patients who presented within 6 mo of symptom onset had normal eosinophil concentrations.

Aside from inconsistencies between the pathology of Löffler's and EMF, the mismatch between the geography of EMF and the ubiquity of parasite-induced eosinophilia calls into question the relationship between these entities [71]. Despite the burden of tropical pulmonary eosinophilia on the basis of lymphatic filariasis in Southeast Asia, for example, these countries have not reported much EMF. Rural Haiti has not reported any EMF cases, despite an active echocardiography service at Deschapelles in the Artibonite Valley [72]. Ecological research of disease causation leaves much room for confounding.

Some have sought a more direct connection between EMF and malarial infection. EMF cases in Uganda have disproportionately come from Rwanda-Burundi immigrant families [73]. While the poverty of these migrants confounds association, others have suggested that movement from zones of lower to higher malaria prevalence might hold the key. Following van der Geld, during the late 1960s Shaper and colleagues found increased levels of anti-malarial and anti-heart antibodies among these migrants and EMF cases in particular [74],[75],[76]. Ziegler, Patel, and colleagues identified a series of familial cases of EMF among Rwanda-Burundi migrants who had massive splenomegaly, a condition associated with malaria-induced immune hyper-reactivity [68],[77]. While the prevalence of plasmodial species does not match the geographic distribution of EMF, these findings point to changes in immunity as a possible pathway from malaria to endocardial disease.

In a separate line of inquiry in Kerala State, India, the high prevalence of EMF along a coastal zone free of filariasis has led investigators to pursue a geochemical hypothesis. Valiathan and Kartha have speculated that cerium or thorium present in monazite deposits may explain regional variation in EMF prevalence in this region [23],[78]. No empirical studies have yet come forward to support this theory.

Investigations into nutritional factors in EMF have focused on a possible connection with cassava toxicity. A case-control study from Uganda has shown an association between EMF and markers of poverty such as farming, lack of shoes, and cassava-based diets with little animal protein [30]. Some have suggested that cerium-mediated cassava toxicity in the setting of protein deficiency may play a role in the pathogenesis of EMF [79]. Despite the known role of cyanogens from improperly processed cassava in konzo, an upper-motor neuron disease reported from Central and East Africa, cardiac manifestations have not had a part in these outbreaks [80].

## Future Directions

Given the difficulty of cardiovascular research in resource-poor settings, the supply of theories about EMF has exceeded the reach of investigation. The disease accounts for a striking proportion of heart failure in some regions. The dissemination of echocardiography in tropical countries should facilitate prospective studies that clarify case definition and generate new insights into the mechanisms of heart failure in sub-Saharan Africa. At the same time, molecular techniques could bring new life to old ideas.

The fusion protein FIP1L1-PDGFRα, a constitutively activated tyrosine kinase found in as many as half of those with the idiopathic hypereosinophilic syndrome, has emerged as a therapeutic target for imatinib [81]. The prevalence of FIP1L1-PDGFRα among those with EMF could give another important clue about the etiology and treatment of this disease.

Studies that measure levels of markers, such as C-reactive peptide or inflammatory cytokines such as tumor necrosis factor α, could help explore the role of inflammation in EMF and suggest therapeutic strategies in early forms of the disease [82].

Echocardiographic studies of patients with hyper-reactive malarial splenomegaly could shed light on the prevalence of early endocardial disorders in this population.

The recent finding that serotonin acts as a chemotactic factor for eosinophils may reignite inquiries into the role of this pathway in EMF [83]. Zanettini and colleagues have found that some anti-Parkinson medications induce valvular fibrosis via their action on 5HT_2B_ receptors [84]. Could polymorphisms in this receptor influence susceptibility to EMF in the presence of intermittent eosinophilia?

**Box 1. Key Learning Points**

- Endomyocardial fibrosis, a restrictive cardiomyopathy, has a high prevalence in tropical regions of sub-Saharan Africa, South Asia, and South America.
- We found no conclusive evidence that parasite-induced eosinophilia explains the pathogenesis of this condition, but the etiological role of eosinophils remains an open question.
- Uncertainty continues about the distribution and causes of the disease.

**Box 2. Five Key Papers in the Field**

1. Davies JNP (1948) Endomyocardial fibrosis in Uganda. East Afr Med J 25: 10–16.
2. Parry EH, Abrahams DG (1965) The natural history of endomyocardial fibrosis. Q J Med 34: 383–408.
3. Connor DH, Somers K, Hutt MS, Manion WC, D'Arbela PG (1967) Endomyocardial fibrosis in Uganda (Davies' disease). 1. An epidemiologic, clinical, and pathologic study. Am Heart J 74: 687–709.
4. Andy JJ, Ogunowo PO, Akpan NA, Odigwe CO, Ekanem IA, et al. (1998) Helminth associated hypereosinophilia and tropical endomyocardial fibrosis (EMF) in Nigeria. Acta Trop 69: 127–140.
5. Rutakingirwa M, Ziegler JL, Newton R, Freers J (1999) Poverty and eosinophilia are risk factors for endomyocardial fibrosis (EMF) in Uganda. Trop Med Int Health 4: 229–235.

## Supporting Information

Video S1 Echocardiogram in a 25 year-old man with predominantly right ventricular EMF from eastern Rwanda. Apical four-chamber view. Note the marked dilatation of the right atrium.
(2.01 MB CDR)
